# Feasibility and efficacy of adding high-intensity interval training to a multidisciplinary lifestyle intervention in children with obesity—a randomized controlled trial

**DOI:** 10.1038/s41366-024-01645-w

**Published:** 2024-10-10

**Authors:** Charlotte Nørkjær Eggertsen, Ryan Godsk Larsen, Kirsten Duch, Morten Bilde Simonsen, Cecilie Brøns Christensen, Tine Caroc Warner, Jens Brøndum Frøkjær, Aase Handberg, Theresa Stjernholm, Esben Thyssen Vestergaard, Søren Hagstrøm

**Affiliations:** 1 Department of Pediatrics and Adolescent Medicine, Aalborg University Hospital, Aalborg, Denmark; 2 Steno Diabetes Center North Denmark, Aalborg University Hospital, Aalborg, Denmark; 3 Department of Clinical Medicine, Aalborg University Hospital, Aalborg, Denmark; 4 ExerciseTech, Department of Health Science and Technology, Aalborg University, Aalborg, Denmark; 5 Research Data and Biostatistics, Aalborg University Hospital, Aalborg, Denmark; 6 Center for Mathematical Modeling of Knee Osteoarthritis, Aalborg University, Aalborg, Denmark; 7 Department of Materials and Production, Aalborg University, Aalborg, Denmark; 8 Department of Pediatrics and Adolescent Medicine, North Denmark Regional Hospital, Hjørring, Denmark; 9 Department of Radiology, Aalborg University Hospital, Aalborg, Denmark; 10 Department of Clinical Biochemistry, Aalborg University Hospital, Aalborg, Denmark; 11 Department of Pediatrics and Adolescent Medicine, Aarhus University Hospital, Aarhus, Denmark; 12 Department of Clinical Medicine, Aarhus University, Aarhus, Denmark

**Keywords:** Paediatrics, Randomized controlled trials, Risk factors

## Abstract

**Background:**

Multidisciplinary lifestyle interventions for children with obesity in Denmark often include recommendations regarding physical activity, but no structured exercise program. We hypothesized that adding high-intensity interval training (HIIT) to a multidisciplinary lifestyle intervention would improve BMI z-score (primary outcome), waist circumference, blood pressure, and health-related quality of life (HRQOL).

**Methods:**

This randomized controlled trial included 173 children and adolescents with obesity. Participants were allocated to 12-months lifestyle intervention (*N* = 83), or 12-month lifestyle intervention accompanied by a 12-week HIIT program (*N* = 90). HIIT consisted of three weekly sessions and included activities eliciting intensities >85% of maximal heart rate.

**Results:**

Attendance rate for the 3-months HIIT intervention was 68.0 ± 23.2%. Dropout was lower in HIIT compared to control at three months (7.8% vs. 20.5%) and 12 months (26.5% vs 48.2%). Changes in BMI z-score did not differ between HIIT and control at 3 months (Mean Difference (MD): 0.01, 95% confidence interval (CI): −0.09; 0.12, *P* = 0.82) or 12 months (MD: 0.06, CI: −0.07;0.19, *P* = 0.34). Across randomization, BMI z-score was reduced by 0.11 (CI: 0.17; 0.06, *P* < 0.01) at 3 months and 0.20 (CI: 0.26;0.14, P < 0.01) at 12 months. At 3 months, HIIT experienced a greater increase in HRQOL of 2.73 (CI: 0.01;5.44, *P* = 0.05) in PedsQL Child total-score and 3.85 (CI: 0.96; 6.74, P < 0.01) in psychosocial health-score compared to control. At 12 months, PedsQL Child physical-score was reduced by 6.89 (CI: 10.97; 2.83, *P* < 0.01) in HIIT compared to control. No group differences or changes over time were found for waist circumference or blood pressure.

**Conclusion:**

Adding a 12-week HIIT program did not further augment the positive effects of a 12-month lifestyle intervention on BMI z-score. Adding HIIT improved HRQOL after 3 months, but reduced HRQOL at 12 months. Implementation of HIIT in community-based settings was feasible and showed positive effects on adherence to the lifestyle intervention.

## Introduction

Childhood obesity is a worldwide epidemic affecting both physical and mental health.

In Denmark, up to 16% of children and adolescents suffer from overweight or obesity [1]. Children and adolescents with obesity are at increased risk of metabolic complications, such as pre-diabetes [2], hypertension [3], dyslipidaemia [4], and excess fat deposition in the liver [5]. In addition, psychological problems, including low quality of life and anxiety, are prevalent in this group of children and adolescents [6, 7]. Childhood obesity often persists into adulthood, increasing the risk of cardiovascular diseases, type 2 diabetes, and various forms of cancer [8–10]. Most obesity-related complications may be reversible if weight status is reduced before puberty and early adulthood [9], which underlines the need for effective treatment strategies targeting children and adolescents with obesity to mitigate the development of later morbidity and mortality.

The Children’s Obesity Clinic´s Treatment (TCOCT) is a family-based multidisciplinary obesity treatment used in Denmark [11]. This approach comprises a lifestyle intervention addressing several aspects of everyday life, including recommendations on frequency and content of meals, physical activity, sleeping patterns, and psychosocial factors related to childhood obesity [11]. The TCOCT protocol improves BMI z-score, metabolic and cardiovascular parameters, and health-related quality of life (HRQOL) in both primary and tertiary care settings [4, 12–16]. Notably, while physical activity is recommended, structured exercise is not an integrated element of the TCOCT protocol.

Physical inactivity is an emerging problem among children and adolescents. In Denmark, only 26% of 11 to 15-year-old adolescents meet the recommended 60 min of moderate-to-vigorous physical activity daily [17], and children and adolescents with obesity are often less active compared with their normal-weight peers [18].

Several studies have provided evidence indicating that high-intensity interval training (HIIT) reduces body weight and improves cardiovascular health and quality of life [19–22]. In combination with nutrition advice, HIIT has proven superior to moderate-intensity continuous training for improving cardiorespiratory fitness in children and adolescents with obesity [23]. Similarly, in combination with multidisciplinary obesity treatment, a recent study in adolescents demonstrated that HIIT, was more effective in reducing BMI z-score and fat mass than traditional training [24]. Despite these health benefits of HIIT, studies have questioned if HIIT is feasible and suitable as an exercise intervention for children and adolescents with obesity [25]. Especially, concerns about the effect of HIIT on affective responses and psychological health parameters, which could impact the implementation as a public health strategy, have been raised [25, 26]. Therefore, this randomized controlled trial aimed to examine the efficacy and feasibility of adding a specialized community based HIIT intervention to a multidisciplinary lifestyle intervention program (TCOCT) in children and adolescents with obesity. Our primary objective was to test the hypothesis that children and adolescents receiving HIIT in addition to TCOCT would experience greater reduction in BMI z-score compared to TCOCT. Secondary objectives were to examine changes in waist circumference, blood pressure, and HRQOL from baseline to 3 and 12-month follow-up between groups. Feasibility was assessed using rates of attendance (HIIT group) and dropout (both groups).

## Subjects and methods

### Study design and population

**Fig. 1:**
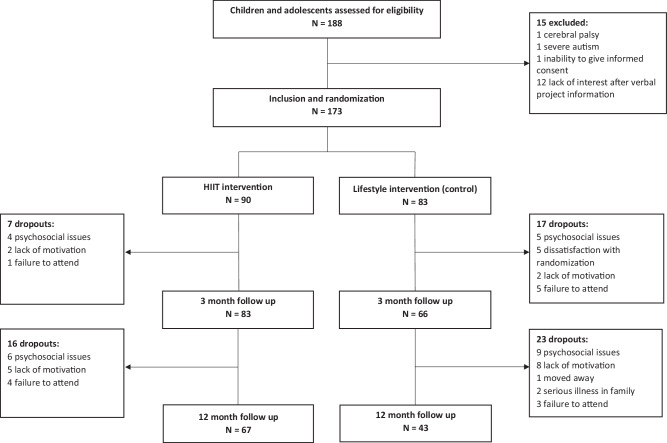
CONSORT flowchart. Flowchart showing participation through the trial.

**Fig. 2:**
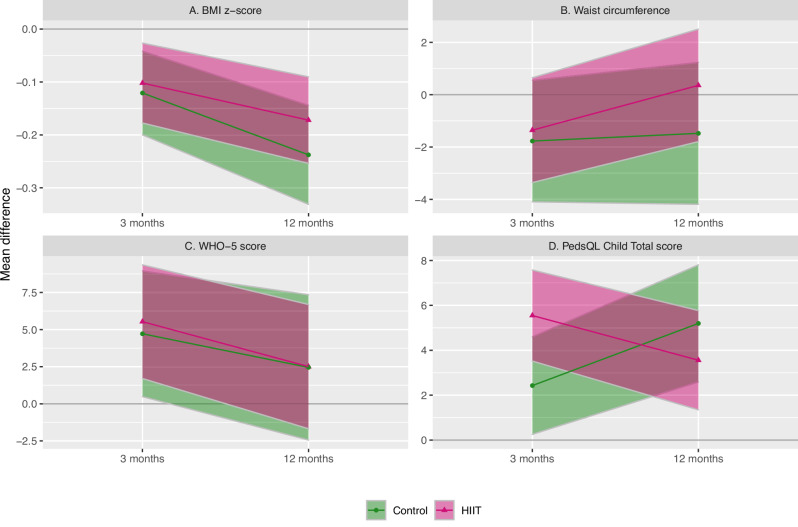
Mean difference between groups from baseline to 3 and 12-month follow-up, using baseline values as references. Shaded area represents 95% confidence intervals. Abbreviations: BMI: Body Mass Index, WHO-5: WHO-Five Well-Being Index, PedsQL: Pediatric Quality of Life Inventory, version 4.0 Generic Core Scales.

This study used a two-armed randomized controlled trial. At baseline, participants were randomized 1:1 to 12 months of TCOCT (control group) or 12 months of TCOCT with 3 months supplementary HIIT (HIIT group) (Fig. 1). We choose to include endpoints for measurements at 3 and 12 months as used in similar studies [27, 28]. In addition, a feasibility study from our group (unpublished), showed that 3 months of HIIT (in combination with TCOCT) was more effective in reducing BMI z-score than 3 months of moderate-intensity training (in combination with TCOCT). This group difference in BMI z-score was maintained at 12 months follow-up, providing a rationale for expecting an effect extending beyond the 3-month HIIT intervention (Fig. 2).

Randomization was conducted using a computerized randomization procedure operated by a third party (Dep. Research and Statistics, Aalborg University Hospital) and using blocks of 4 to 6 participants, with individuals stratified on local municipality.

Participants were recruited from 6 municipal obesity clinics in Northern Denmark and the outpatient clinic for childhood obesity treatment, Department of Pediatrics and Adolescent Medicine, Aalborg University Hospital. Inclusion criteria were BMI > 90 percentile for age and sex by WHO growth charts 2007 [29], and age 9 to 16 years.

Children and adolescents with physical limitations (e.g. bone fractures, heart or lung disease, or metabolic disease) or mental illness, which would complicate participation in the 12 weeks HIIT intervention, were not included. Children and adolescents with minor disabilities (e.g., well-treated ADHD) who were able to take part in the HIIT intervention were included in order to embrace the heterogeneity and vulnerability of the group of children and adolescents with obesity.

The trial followed the ethical guidelines of the Declaration of Helsinki and was approved by the North Denmark Region (ID: 2020-173) and the North Denmark Region Committee on Health Research Ethics (ID: N-20200035). The project was reported according to CONSORT 2010 statement and was registered on ClinicalTrials.gov (ID: NCT05465057). Informed consent was signed by parents or legal guardians before participation.

### Lifestyle intervention

All enrolled children and adolescents were initially introduced to the TCOCT protocol [11]. Each participant was scheduled for follow-up visits in the outpatient or municipal obesity clinic by a trained pediatric nurse and dietician every 6 to 12 weeks. Based on information obtained from interview and physical examination, an individual treatment plan (consisting of 15 to 20 items, Supplementary Fig. 1) was outlined in collaboration with the family in accordance with the TCOCT protocol, allowing individual needs of each child/family to be included in the treatment plan [11].

### HIIT intervention

The HIIT protocol consisted of three weekly sessions of 45–50 min and was conducted in groups of 6–10 children and adolescents. The training sessions were conducted in the participant’s local environment (school facilities). The sessions were primarily conducted indoors, but due to national restrictions during the COVID-19 pandemic, five teams (*n* = 33; 37%) conducted the sessions outdoors for four weeks. Indoor and outdoor training sessions comprised the same activities (examples provided in Supplementary Fig. 2). In general, the COVID-19 lockdown led to a three-month halt in recruitment, and an 8–10-week postponement of HIIT sessions following baseline measurements. To account for potential changes in weight and height during this period, additional anthropometric measurements were collected at the initiation of the HIIT intervention for the five teams affected.

All training sessions were supervised by two sports science or physiotherapy students, who had received training in organizing HIIT activities for children and adolescents with obesity. All HIIT activities were designed to elicit intensities >85% of maximal heart rate in 4 bouts of 4-minute intervals, with 3 min of active recovery between bouts [27]. The activities were tailored to be playful, non-competitive, and comprised strength-based exercise, ball games, or running games. New activities (or adjustment of activities) were introduced regularly to maintain interest and stimulate high intensity of the activities in the HIIT sessions (Supplementary Fig. 2). The intensity level was monitored and quantified using a real-time team heart rate (HR) system with Suunto dual comfort belts (Suunto, Vantaa, Finland) and iQniter cardio training software 3.5 (iQniter, Aalborg, Denmark). Specifically, we recorded HR continuously throughout the duration of each session without segmenting warm-ups, intervals, active breaks, and cool-downs. This approach allowed us to determine minutes spent in high (>85%), moderate (60 to 85%), or low (<60% of maximal HR) for each session. Each participant’s maximal HR was estimated using the revised age-predicted maximal heart rate equation (208–(0.7×age)) [30].

### Measurements

Anthropometrics (height, weight, waist and hip circumference, BMI, BMI z-score), blood pressure, pubertal stage data, and HRQOL were measured at baseline, 3 months, and 12 months.

Height was measured without shoes to the nearest 0.1 cm using a wall-mounted stadiometer, and weight was measured to the nearest 0.1 kg wearing light indoor clothes without shoes using a calibrated scale (Seca 799, Hamburg, Germany, or Tanita DC 360S, Soeborg, Denmark). BMI (kg/m^2^) was converted into BMI z-scores according to the WHO Reference 2007 [29]. Waist circumference was measured to the nearest 0.1 cm with participants standing using stretch-resistant tape at the midpoint level between the lowest rib and the top of the iliac crest. Blood pressure was measured on the right arm after 5 min of rest in a sitting position using an automated blood pressure monitor (Omron M7 Intelli IT, Kyoto, Japan). Blood pressure measurements were conducted three times, and the mean of the three recordings was reported. Pre-hypertension was defined as average systolic and/or diastolic blood pressure ≥ 90th percentile and < 95th percentile, and hypertension was defined as ≥ 95th percentile for sex, age, and height according to the AAP guidelines [31].

Puberty status was assessed using Tanner’s Pubertal Scale images presented to the child, scored 1–5 [32, 33]. Girls pointed out the image best corresponding to their stage of breast development, whereas boys pointed out the image best corresponding to their development of external genitalia. Based on the score, the following categories were subsequently defined: ’Pre-pubertal‘(Tanner 1), ’Early puberty‘(Tanner 2-3) and ’Late puberty’ (Tanner 4-5).

Psychological well-being and HRQOL were measured via validated questionnaires using the Pediatric Quality of Life Inventory, version 4.0 Generic Core Scales (PedsQL) [34] and WHO-Five Well-Being Index (WHO-5) [35, 36].

### Statistics and data analysis

Sample size was calculated using a two-sided t-test for changes in the primary outcome measure, BMI z-score. Based on previous results [14, 37], a power of 80%, a significance level of 5%, a mean difference in BMI z-score of 0.2, and a standard deviation (SD) of 0.45, the total sample size was 162. Accounting for 25% dropout, the required sample size was 202 children and adolescents.

Prior to analysis, all variables were checked for outliers and sample distribution. Baseline values were presented with mean and SD for continuous variables and count and percentage for categorical variables.

A generalized linear mixed model (GLMM) with subjects as random intercept was used to estimate the mean difference (MD) between groups. Both a crude (adjusted for baseline scores) and an adjusted analysis were performed to estimate the effect of the HIIT intervention. The adjusted analysis included baseline score, puberty category, and sex. A GLMM was also used to examine overall changes over time. If difference between groups was observed, within-group changes were also reported. Missing values were handled by GLMM.

Participants with a baseline measurement and at least one follow-up measurement were included in the analysis.

For the HIIT group only, the attendance rate of ≥70% was accessed using a GLMM for both 3 and 12 months. Attendance rate was calculated as (number of sessions attended/total sessions offered)×100.

All analyses are presented with 95% confidence intervals (CI) and a significance level of 5%. Analysis was done with STATA 18 (Stata Corp LLC, TX, USA).

Several sensitivity analyses were performed: (i) missing values were treated as last value carried forward and as complete case analysis, (ii) overall effect of sex and puberty category was assessed for each visit and outcome measure using a GLMM, (iii) a generalized linear model (GLM) was used to calculate the relative risk (RR) and odds ratio (OR) of dropout at 3 months based on baseline measures for each intervention group, (iv) a yes/no variable indicating if participant’s training sessions were affected by the COVID-19 lockdown was added to the adjusted analysis, and the overall effect of the lockdown was assessed for each outcome measure.

## Results

### Recruitment and dropout

A total of 173 children and adolescents (101 boys, 12.3 ± 1.7 years) with a BMI z-score of 2.5 ± 0.6 participated in the study from October 2020 to May 2023. Baseline characteristics are presented in Table 1. A CONSORT diagram (Fig. 1) summarizes the participant flow through each trial stage.

**Table 1:** Baseline characteristics.

	HIIT	Control	Total	Missing
	*N* = 90	*N* = 83	*N* = 173	*N* (%)
Age, mean (SD)	12.3 (1.7)	12.3 (1.8)	12.3 (1.7)	0 (0.0)
Sex: male, n (%)	53 (58.9)	48 (57.8)	101 (58.4)	0 (0.0)
Weight, mean (SD) kg	73.3 (19.1)	69.3 (18.3)	71.4 (18.8)	0 (0.0)
Height, mean (SD) m	1.6 (0.11)	1.6 (0.11)	1.6 (0.11)	0 (0.0)
Waist, mean (SD) cm	93.3 (12.5)	91.7 (11.9)	92.5 (12.2)	2 (1.2)
BMI, mean (SD) kg/m^2^	28.3 (4.6)	27.2 (4.5)	27.8 (4.5)	0 (0.0)
BMI z-score, mean (SD)	2.6 (0.6)	2.4 (0.6)	2.5 (0.6)	0 (0.0)
Systolic blood pressure, mean (SD) mmHg	107.9 (10.9)	105.8 (9.6)	106.9 (10.4)	1 (0.6)
Diastolic blood pressure, mean (SD) mmHg	67.8 (7.3)	67.1 (7.3)	67.5 (7.3)	1 (0.6)
Puberty group, n (%)				5 (2.9)
Pre-puberty	18 (20.9)	16 (19.5)	34 (20.2)	
Early puberty	38 (44.2)	39 (47.6)	77 (45.8)	
Late puberty	30 (34.9)	27 (32.9)	57 (33.9)	
WHO-5 score, mean (SD)	66.8 (15.6)	65.1 (16.4)	66.0 (16.0)	2 (1.2)
PedsQL Child				
Total score, mean (SD)	73.0 (13.0)	72.7 (12.3)	72.9 (12.7)	5 (2.9)
Psychosocial score, mean (SD)	69.7 (14.1)	69.7 (13.1)	69.7 (13.6)	5 (2.9)
Physical score, mean (SD)	79.2 (15.0)	78.3 (14.0)	78.8 (14.5)	1 (0.6)
PedsQL Parents				
Total score, mean (SD)	71.4 (13.3)	69.4 (13.4)	70.4 (13.4)	4 (2.3)
Psychosocial score, mean (SD)	68.4 (14.2)	66.1 (14.0)	67.3 (14.1)	4 (2.3)
Physical score, mean (SD)	76.8 (14.9)	74.5 (16.9)	75.7 (15.8)	2 (1.2)

*SD* standard deviation, *BMI* body mass index, *WHO-5* WHO-Five Well-Being Index, *PedsQL* Pediatric Quality of Life Inventory, version 4.0 Generic Core Scales.

During the 12-week intervention, the HIIT group experienced a dropout rate of 7.8% (CI: 2.3%; 13.3%), while the control group experienced a dropout rate of 20.5% (CI: 11.8%; 29.2%). Dropout in the HIIT group occurred particularly in the first two weeks of training (1 child and 3 adolescents) and after the COVID-19 lockdown (3 adolescents). At 12 months follow-up, the HIIT group experienced a 25.5% (CI: 16.5%; 34.5%) dropout, while the control group experienced a dropout rate of 48.2% (CI: 37.5%; 58.9%).

At 3 months, a one-unit higher BMI z-score resulted in a higher risk for dropout in the control group (RR: 2.20, CI: 0.96; 5.03, corresponding to an OR: 2.83, CI: 1.04; 7.71) and a lower risk for dropout in the HIIT group (RR: 0.39, CI: 0.12; 1.30, corresponding to an OR: 0.34, CI: 0.09; 1.30).

### Training intensity and attendance

A total of 90 children and adolescents participated in the HIIT intervention and showed an average attendance rate of 68.0 ± 23.2% over the 12-week intervention. Fifty-five (61.1%) children and adolescents completed ≥70% of the HIIT sessions.

The average exercise session lasted 47.5 ± 10.5 min, and the participants spent on average 8.7 ± 4.8 min in high intensity, 31.5 ± 4.2 min in moderate intensity, and 6.7 ± 5.0 min in low intensity.

### BMI z-score and waist circumference

We found no difference in BMI z-score between HIIT and control at 3 months (MD: 0.01, CI: −0.09; 0.12) or 12 months (MD: 0.06, CI: −0.07; 0.19). Similarly, no difference between groups were seen in waist circumference at 3 months (MD: 1.34 cm, CI: −1.58; 4.26 cm) or 12 months (MD: 2.81 cm, CI: −0.51; 6.12 cm) (Table 2). Adjustment and sensitivity analysis did not change the results (not shown).

**Table 2:** Effect of HIIT intervention (crude), mean difference with 95% confidence interval between the intervention group (HIIT) and control group (control).

	Participants (N)	Mean difference 3 months (95% CI)	*P* value	Particpants (N)	Mean difference 12 months (95% CI)	*P* value
BMI z-score	152	0.01 (−0.09; 0.12)	0.82	107	0.06 (−0.07; 0.19)	0.34
Waist (cm)	150	1.34 (−1.58; 4.26)	0.37	104	2.81 (−0.51; 6.12)	0.09
Systolic blood pressure (mmHg)	149	0.05 (−2.62; 2.73)	0.97	101	−0.33 (−3.39; 2.72)	0.83
Diastolic blood pressure (mmHg)	149	−0.72 (−2.67; 1.22)	0.47	101	−0.51 (−2.73; 1.72)	0.66
WHO-5 score	149	1.49 (−3.56; 6.54)	0.56	101	0.68 (−5.27; 6.64)	0.82
PedsQL Child						
Total score	143	2.73 (0.01; 5.44)	0.05	92	−2.03 (−5.25; 1.18)	0.22
Psychosocial score	143	3.85 (0.96; 6.74)	0.01	92	0.89 (−2.49; 4.27)	0.61
Physical score	147	0.87 (−2.59; 4.32)	0.62	102	−6.89 (−10.97; −2.83)	<0.01
PedsQL Parents						
Total score	145	1.78 (−1.29; 4.86)	0.26	96	−3.11 (−6.66; 0.45)	0.09
Psychosocial score	145	1.21 (−2.11; 4.54)	0.47	96	−2.59 (−6.43; 1.23)	0.18
Physical score	147	2.46 (−1.35; 6.28)	0.21	99	−5.04 (−9.53; −0.56)	0.03

*SD* standard deviation, *BMI* Body Mass Index, *WHO-5* WHO-Five Well-Being Index, *PedsQL* Pediatric Quality of Life Inventory, version 4.0 Generic Core Scales.

Across randomization (pooled data), BMI z-score was reduced by 0.11 (CI: 0.17; 0.06) at 3 months and by 0.20 (CI: 0.26; 0.14) at 12 months. There were no significant changes in waist circumference at 3 and 12 months (Table 3).

**Table 3:** Overall improvement from baseline to 3 and 12-month visits, mean difference with 95% confidence interval (pooled cohort).

	Participants (N)	Mean difference Baseline to 3 months (95% CI)	*P* value	Participants (N)	Mean difference baseline to 12 months (95% CI)	*P* value
BMI z-score	152	−0.11 (−0.17; −0.06)	<0.01	107	−0.20 (−0.26; −0.14)	<0.01
Waist (cm)	150	−1.54 (−3.06; 0.02)	0.05	104	−0.36 (−2.06; 1.33)	0.67
Systolic blood pressure (mmHg)	149	0.27 (−1.20; 1.74)	0.72	101	1.91 (0.29; 3.52)	0.02
Diastolic blood pressure (mmHg)	150	−0.64 (−1.82; 0.54)	0.29	101	0.57 (−0.71; 1.86)	0.38
WHO-5 score	149	5.18 (2.34; 8.02)	<0.01	101	2.47 (−0.71; 5.65)	0.13
PedsQL Child						
Total score	143	4.18 (2.67; 5.69)	<0.01	92	4.09 (2.38; 5.81)	<0.01
Psychosocial score	143	3.93 (2.34; 5.51)	<0.01	92	3.77 (1.98; 5.57)	<0.01
Physical score	147	4.98 (2.86; 7.09)	<0.01	102	4.69 (2.33; 7.06)	<0.01
PedsQL Parents						
Total score	145	5.52 (3.86; 7.19)	<0.01	96	3.86 (1.99; 5.73)	<0.01
Psychosocial score	145	5.62 (3.82; 7.43)	<0.01	96	3.87 (1.85; 5.89)	<0.01
Physical score	147	5.79 (3.59; 7.99)	<0.01	99	4.49 (2.03; 6.95)	<0.01

*SD* standard deviation, *BMI* Body Mass Index, *WHO-5* WHO-Five Well-Being Index, *PedsQL* Pediatric Quality of Life Inventory, version 4.0 Generic Core Scales.

### Blood pressure

No significant differences in diastolic or systolic blood pressure were found between groups (Table 2). Across randomization, there were no changes in diastolic blood pressure at 3 or 12 months (Table 3). However, systolic blood pressure was increased after 12 months (Table 3).

At baseline, 4.6% of the children and adolescents had elevated systolic blood pressure (pre-hypertension) and 4.6% had hypertension. For diastolic blood pressure, 6.9% of the cohort had pre-hypertension and 7.5% had hypertension at baseline. There were no significant changes in the distribution of these blood pressure categories at 3 or at 12 months.

### Quality of life

There were no differences between groups in WHO-5 well-being index after 3 or 12 months. The cohort showed an increase in WHO-5 score at 3 months, which did not sustain at 12 months (Table 3).

At 3 months, the increase in HRQOL was greater in the HIIT group, with a mean difference in PedsQL child self-report, total score of 2.73 (CI: 0.01; 5.44), and a mean difference in psychosocial health score of 3.85 (CI: 0.96; 6.74) compared to the control group (Table 2). Within-group analysis showed an increase in PedsQL child self-report, total score of 5.55 (CI: 3.52; 7.58) in the HIIT group and 2.43 (CI: 0.25; 4.6) in the control group. The increase in psychosocial health score was 5.88 (CI: 3.72; 8.05) in the HIIT group and 1.42 (CI: −0.78; 3.62) in the control group.

At 12 months, the control group experienced a greater increase in PedsQL child self-report physical score than the HIIT group (Table 2). The increase in physical score was 9.14 (CI: 5.51; 12.77) in the control group and 1.86 (CI: −1.14; 4.87) in the HIIT group.

The PedsQL parents proxy report showed higher scores, yet not significant, in all three domains in the HIIT group at 3 months (Table 2). At 12 months, physical scores were greater in the control group compared with the HIIT group (Table 2).

### Sensitivity analysis

Sensitivity analysis showed a larger mean reduction in BMI z-score in boys compared to girls at 12 months of 0.16 (CI: 0.29; 0.03), corrected for baseline BMI z-score. A greater reduction in diastolic blood pressure was seen in boys compared to girls at 12 months (MD: 3.58, CI: 5.77; 1.39). No differences between sexes were found for waist circumference or systolic blood pressure.

For HRQOL, the WHO-5 index for boys increased more than for girls at 12 months (MD: 7.89; CI: 1.95; 13.84). The PedsQL total (MD: 2.81; CI: 0.08; 5.53) and psychosocial domains (MD: 3.14; CI: 0.23; 6.04) increased for boys compared to girls at 3 months. These differences between sexes were also evident at 12 months in PedsQL total (MD: 4.21; CI: 0.99; 7.44), psychosocial (MD: 4.25; CI: 0.85; 7.64), and physical domains (MD: 4.87; CI: 0.73; 9.01).

Sensitivity analysis for the effect of baseline puberty on the outcome measures at 3 months showed no difference between puberty categories for BMI z-score, diastolic blood pressure or HRQOL (WHO-5 and PedsQL). However, a significant increase in waist circumference of 4.6 cm (CI: 0.93; 8.34 cm) was found at 3 months in the “Early puberty” group compared to pre-pubertal children. A greater increase in systolic blood pressure was found in the groups of “Early puberty” (MD: 3.53; CI: 0.19; 6.87) and “Late puberty” (MD: 4.22; CI: 0.51; 7.92) compared to the group of pre-pubertal children. No differences between puberty categories were found at 12 months for any outcome measures.

Participants who attended ≥70% of the training sessions exhibited greater PedsQL total score and physical score at 3 months than those who attended <70%. There were no other differences between these two sub-groups (Supplementary table 1). Sensitivity analyses for missing observations did not change the results.

Adjusting for COVID-19 lockdown did not affect the group differences and changes over time (data not shown). However, at 3 months, waist circumference was significantly increased among participants whose intervention was affected by COVID-19 compared to those unaffected (MD: 4.1, CI: 1.07; 7.15).

## Discussion

This two-armed randomized controlled trial aimed to examine the feasibility and efficacy of adding a 12-week HIIT program to a 12-month family-based multidisciplinary intervention.

We found no differences in the primary outcome, BMI z-score, between the HIIT and control group. However, across randomization, significant reductions in BMI z-score were found at 3 months and 12 months follow-up. At 3 months, HRQOL was improved in the HIIT group, but reduced below the level of the control group at 12 months. Sensitivity analyses indicated that participation in HIIT resulted in lower dropout and better adherence to the lifestyle intervention.

### Effects of TCOCT and exercise on BMI z-score

Across randomization, we found significant reductions in BMI z-score of 0.11 points at 3 months and 0.20 points after 12 months. In addition, boys experienced a greater reduction in BMI z-score than girls at 12 months. Consistent with these results, a cohort of Danish children admitted to a tertiary obesity clinic, showed reductions in BMI z-score of 0.30 (CI: 0.39; 0.21) in boys and 0.19 (CI: 0.25; 0.13) in girls after one year of TCOCT intervention [12]. Our results thus reinforce that a multidisciplinary family-based lifestyle intervention (i.e., TCOCT) is effective in improving BMI z-score in children and adolescents with obesity, with possible greater benefit for boys. BMI z-score reductions of more than 0.25 points have been associated with improvements in cardiometabolic risk factors [38], but even a reduction in BMI z-score of 0.10 has been associated with health benefits among children with obesity [37].

A novel aspect of our study was to examine if adding a 3-month HIIT protocol to the TCOCT intervention would augment a reduction in BMI z-score at 3- or 12-months follow-up. We found no differences between groups. It is possible that the short duration of the HIIT intervention provided insufficient stimulus for eliciting changes in body composition. However, the lack of additive effects of HIIT is consistent with previous studies reporting only modest effects of 12 weeks of HIIT on BMI z-score and waist circumference among children and adolescents with obesity [23, 39]. A recent study (EFRIGO) that included a 6-month supplementary HIIT intervention to a multidisciplinary lifestyle intervention showed no difference in BMI between groups. The EFIGRO study used a longer exercise intervention (22 weeks) and a larger weekly training volume (3 sessions of 90 min) than the present study [40]. Taken together, these results suggest that adding exercise to multidisciplinary lifestyle interventions do not always promote greater changes in BMI z-score in children and adolescents with obesity [41]. Notably, the EFIGRO study reported a greater reduction in hepatic fat and LDL cholesterol in the HIIT group compared with the control group (lifestyle intervention only) [40]. Consistent with these findings, a Danish study reported metabolic improvements in 80% of the children participating in a lifestyle intervention despite no change in BMI z-score [42]. These results suggest that multidisciplinary lifestyle intervention, including recommendations about physical activity or an exercise program, may elicit metabolic improvements, even without marked reductions in BMI z-score.

### Effects of TCOCT and HIIT on blood pressure

We found no clinically relevant changes in blood pressure. At baseline, the majority of the participants had blood pressure within the normal range, which limits the potential for improvements in response to the interventions. Less than 8% of the participants showed signs of hypertension, which is lower than the prevalence of 16% reported by Hvidt et al. in a comparable cohort of Danish children and adolescents with obesity [3].

### Effects of lifestyle interventions and HIIT on HRQOL

The physiological links between obesity-related risk behaviors, including physical inactivity and psychosocial health, are well-known and assumed to be bi-directional [43]. Studies have shown an inverse relationship between obesity and well-being on both physical and psychosocial parameters [6]. A novel result from the present study was that adding a 3-month HIIT program to the TCOCT protocol resulted in a greater improvement in HRQOL after 3 months, measured by PedsQL. Specifically, the HIIT group reported a greater increase in PedsQL child total score and psychosocial health score. Furthermore, the boys experienced overall greater improvement in HRQOL than the girls. These findings extend results from previous studies investigating the effects of exercise interventions on HRQOL [44]. Goldfield et al. reported an increase in PedsQL child total score of 5.5 (CI: 1.4; 9.6) after a 22-week combined lifestyle and aerobic exercise intervention with 4 weekly training sessions compared to control groups [45]. Together, these results emphasize the relevance of including physical activity programs as a central element in obesity treatment interventions.

Consistent with Goldfield et al. [45], our sensitivity analysis showed that participants with ≥70% attendance in HIIT sessions exhibited a greater increase in HRQOL, particularly in the physical score. This is an important finding because enjoyment and positive feelings of competence and confidence are the main psychological drivers of engagement and adherence to physical activities [46, 47]. A 4-month HIIT-centered multidisciplinary intervention in adolescents with obesity showed improvement in the physical dimension of HRQOL, with no effects on the mental dimensions [48]. It is interesting to note that HIIT interventions conducted in different studies appear to improve different aspects of HRQOL and that these effects may even vary by sex. Future studies are required to investigate the interplay between HIIT (or exercise) interventions, sex, and HRQOL in children and adolescents with obesity. In turn, this information may help to improve mental health factors in this group of vulnerable children and adolescents [49].

At 12 months, we found lower levels of HRQOL in the HIIT group compared to the control group. One explanation could be the influence of the COVID-19 pandemic and related restrictions in the society, which lasted until the beginning of 2022. A study reported that participation in physical activity in relation to school and regular sport clubs was significantly reduced during the COVID-19 lockdowns and still showed a lower level in the summer of 2022 [50]. Another explanation could be that termination of the 3-month HIIT program left many of the HIIT participants with a feeling of sadness and abandonment. This interpretation is supported by feedback from the HIIT participants and their families, who noted that they could not find a suitable exercise program to replace the HIIT. This emphasizes the importance of implementing permanent exercise programs tailored to this population. Implementing such programs in local communities should consider the cost related to the use of facilities, training equipment, and educated trainers. In our study, two trainers were allocated to each team of 6–10 participants.

### HIIT intervention

The attendance rate for HIIT sessions reached 68% across the 3 months, which is high compared to reports from similar studies [51, 52]. In a 6-months HIIT intervention in adolescents with overweight, Herget et al. reported that the attendance rate declined from 75% in the first two months to 15% in the last three months [52]. The dropout rate of 7.8% in our HIIT group during the first three months was lower than the dropout rate in the control group (20.5%). Notably, the dropout rate at 12 months was also lower in the HIIT group (25.5%) compared to the control group (48.2%). Jørgensen et al reported a dropout rate of 43% after 12-month TCOCT protocol in a similar cohort of Danish children with obesity [13]. Indeed, the lower dropout rates in the HIIT group compared to the control group indicate that participation in a tailored exercise program increases adherence to a 12-month TCOCT protocol. In addition, our analyses revealed that a one-unit higher BMI z-score in the control group resulted in a higher RR for dropout after 3 months, which was not seen in the HIIT group. This result supports that exercise programs may be a viable option in reducing attrition in programs focused on treating obesity, particularly for children and adolescents with very high BMI z-scores.

While puberty is known to be associated with increasing BMI z-score [53] and decreasing HRQOL [54], our sensitivity analysis showed no effect of pubertal category on BMI z-score or HRQOL. Hence, these results suggest that the effects of the interventions (TCOCT and HIIT) were not different across the three puberty categories. However, there were effects of puberty stage on changes (from baseline to 3 months) in waist circumference and systolic blood pressure, such that ‘Early puberty’ (for waist circumference and systolic blood pressure) and ‘Late puberty’ (systolic blood pressure) showed greater increases compared to pre-pubertal children.

Adolescence is known to be the life stage with the biggest dropout in sports and physical activity programs [46, 53]. Psychological issues and concerns about appearance have been found to predict low adherence to training interventions in adolescents with obesity [51]. Enjoyment, family support, creating social relations, and full participation in age-appropriate activities are important elements for children and adolescents in order to overcome challenges in relation to physical activities [54]. The fact that our HIIT intervention was created with a playful, supportive, and non-competitive perspective through various modalities may have positively influenced adherence and dropout rates. These results support the importance of establishing tailored training interventions that embrace a safe and non-stigmatizing environment with enjoyable and motivating activities to ensure long-term adherence for children and adolescents. In addition, taking advantage of continuous HR monitoring allowed us to demonstrate that the HIIT sessions were effective in eliciting 40 min of moderate-to-high intensity physical activity (>60% maximal HR) which constitutes a large proportion of recommended daily physical activity.

### Strengths and limitations

A limitation of the study is that we only enrolled and randomized 173 participants, which is fewer than the estimated number from the sample size calculation (*N* = 202). Our study was conducted from 2020–2023, and the intervention (including recruitment) was challenged by the COVID-19 pandemic and lockdown periods in the winter of 2020/2021. We experienced a general reluctance to participate in the intervention after the COVID-19 lockdown, which hindered recruitment for the study. The sensitivity analysis showed that COVID-19 did not affect the difference across intervention but might have decreased the signal from the intervention. This interpretation is supported by recent studies reporting increases in BMI z-score and reduced HRQOL in school children after the COVID-19 lockdowns [50, 55]. Notably, COVID-19 affected participants in our study experienced a significant increase in waist circumference at 3 months. This could have been mediated by lower levels of habitual physical activity and increased sedentary behavior during the lockdown periods, as reported in other studies [56, 57]. A recent review supports this interpretation by reporting a global reduction of 20% in daily physical activity among children and adolescents during the lockdown, with an even greater reduction in activities with higher intensity [58].

A strength of our study is the randomized design, which minimized the risk of selection bias. In addition, we developed a HIIT program that used a variety of activities, which allowed the trainers to adjust the training according to the children’s preferences, skills, and competencies. This concept also allowed children and adolescents with minor psychological disabilities (such as well-treated ADHD) to participate. Indeed, this approach embraces the great heterogeneity in the population of children and adolescents with obesity. It is possible that this approach could have reduced the intensity of the HIIT sessions. However, in line with findings from a recent study of adolescent school children [56], we found that this approach increased participants’ motivation and enjoyment of the HIIT activities.

In conclusion, our results show that it is feasible to implement 3 months of HIIT in a community-based setting tailored for children and adolescents with obesity. In fact, participation in the HIIT program appeared to promote greater adherence to the lifestyle intervention, particularly for those with severe obesity. Addition of supervised HIIT training to the TCOCT protocol did not augment the reduction in BMI z-score. However, HIIT was accompanied by greater HRQOL at 3 months, but lower HRQOL at 12 months, compared with the control group. Overall, the boys experienced greater effects of the intervention on BMI z-score and HRQOL. Future studies should further investigate the potential of using tailored exercise programs in community-based settings to promote greater adherence to lifestyle interventions in children and adolescents with obesity.

## Supplementary information

Supplementary figures and tables

## Data Availability

Data is available upon reasonable request.
