# Supplementary material for: Feasibility and efficacy of adding high-intensity interval training to a multidisciplinary lifestyle intervention in children with obesity—a randomized controlled trial
Source: Int J Obes (Lond). 2024 Oct 10;49(2):269–77. doi: 10.1038/s41366-024-01645-w (PMC11805701; doi:10.1038/s41366-024-01645-w)
Supplement: Supplementary file 1 — Supplementary figures and tables [file 41366_2024_1645_MOESM1_ESM.pdf]

Supplementary figure 1: List of items of advice offered by TCOCT protocol (11).

Breakfast	Lunch	Afternoon	Dinner	Activity	General
Eat breakfast	*Bring* and eat lunch	*Eat* in the afternoon	*Eat* dinner	*Seek* to increase physical activity to one hour daily	*1–2* pieces of fruit per day
Reduce eating at TV/PC	*Reduce* eating at TV/PC	*Reduce* eating at TV/PC	*Reduce* eating at TV/PC	*Decrease* TV/PC to 2 hours	*Reduce* fast food
Reduce toast/white bread	*Reduce* toast/white bread	*Reduce* toast/white bread	*Reduce* toast/white bread	*Decrease* TV/PC to 3 hours	*Fast food* once per month
Reduce fatty/sweet yoghurt	*Reduce* food high in fat and sugar	*Reduce* food high in fat and sugar	*Reduce* food high in fat and sugar	*Reduce* TV/PC before 6 pm	*Reduce* candy
Instead dark bread or cereals high in fibers and low in fat and sugar	*Instead* eat dark bread high in fibers	*Instead* eat dark bread high in fibers	*Instead*, dinner should be low-fat, low in sugar and high in fibers	*Bicycle* to school	*Candy* once a week for $1 (under age 11) or $2 (over age 11)
Reduce chocolate in either drink or on bread	*Reduce* chocolate in either drink or on bread	*Reduce* chocolate in either drink or on bread	*Reduce* chocolate in either drink or on bread	*Dance* to music at home	*Reduce* ice-cream
Reduce jam or honey	*Reduce* jam or honey	*Reduce* jam or honey	*Portions* arranged in the kitchen	*Play*	*Ice cream* once a week
Reduce high-fat cheese, max 13%	*Reduce* high-fat cheese, max 13%	*Reduce* high-fat cheese, max 13%	Normal size of Portions; 1/5 meat/fish, 2/5 potatoes, rice, or pasta, 2/5 salad and vegetables	*Bicycle*	*Reduce* cake
Instead low-fat fish, meat, and vegetables	*Instead* low-fat fish, meat, and vegetables	*Instead* low-fat fish, meat, and vegetables	*Second* portion after 20 minutes	*Gymnastics*	*Cake* once a week
Reduce butter	*Reduce* butter	*Reduce* butter	*Gravy* max 5%	*Swim*	*Reduce* snacking
Instead margarine (max 35% fat)	*Instead* margarine (max 35% fat)	*Instead* margarine (max 35% fat)	*Instead* margarine (max 35% fat)	*Basketball*	*Snacking* once a week
Reduce high-fat milk	*Reduce* high-fat milk	*Reduce* high-fat milk	*Reduce* high-fat milk	*Handball*	*Reduce* soda, juice, iced tea, or cocoa
Reduce soda, juice or iced tea	*Reduce* soda, juice or iced tea	*Reduce* soda, juice or iced tea	*Reduce* soda, juice or iced tea	*Tennis*	*Soda*, juice, iced tea, and cocoa once a week
Instead water or low-fat milk	*Instead* water or low-fat milk	*Instead* water or low-fat milk	*Instead* water or low-fat milk	*Ice-hockey*	*Instead* water or low-fat milk
Reduce candy or snacking	*Reduce* candy or snacking	*Reduce* candy or snacking	Reduce candy or snacking	*Attend* other organized discipline	*Reduce* smoking and alcohol

Supplementary figure 2: Example of activities at a HIIT session.

Activity:	Activity description:	Music / equipment:	Progression / regression:
**Warm up: dodge ball**<br>-with exercises / running<br>- 10-15 minutes<br>- Intensity (60-70 % HRmax)	- The one with the ball tries to hit the rest of the players without walking<br>- When hit, the person performs 10 x repetition of an exercise by own choosing<br>For example: Jumping Jacks, squats, push-ups, crunches, etc.	**Music:**<br>Happy music<br><br>**Equipment:**<br>2 foam balls	**Progression:**<br>- Running laps instead of exercises<br>- The coach decides the exercises and amount<br>- 2 balls at a time<br><br>**Regression:**<br>- Less repetitions of the exercise
**Catch the tail – tag:**<br>- 1 x 4 minutes<br>- Intensity (> 85 % HRmax)	- Every participant gets two "tails" each.<br>- Set a timer for 4 min. The participants have to get as many tails as possible.<br>- With or without a tail of your own – you are still in the game and can catch tails<br>- After 4 min. each participant counts their tails	**Music:**<br>Music with a higher BPM to facilitate running. Preferable music the participants knows.<br><br>**Equipment:**<br>- 2 x cloth or rag per player	**Progression:**<br>- Increase the time playing<br><br>**Regression:**<br>- Decrease the time playing
**Break: 3 minutes rest / active recovery (walking around the gym, moving equipment) / socializing**			
**Soccer:**<br>- Tip over objects<br>- 1 x 4 minutes<br>- Intensity (> 85 % HRmax)	- The players are divided in two teams<br>- Each team places 3 objects in their lane end<br>- To reach a goal each team must tip over the opponent team's objects<br>- There is no permanent goalkeeper	**Music:**<br>Music with high BPM to facilitate higher intensity movement.<br><br>**Equipment:**<br>- 6 objects<br>- 1 Soccer ball	**Progression:**<br>- All team members must touch the ball before reaching for a goal<br>- Constant movement, with and without the ball<br><br>**Regression:**<br>- Reduce the number of objects
**Break: 3 minutes rest / active recovery (walking around the gym, moving equipment) / socializing**			
**Soccer:**<br>- Tip over objects<br>- 1 x 4 minutes<br>- Intensity (> 85 % HRmax)	- The players are divided in two teams<br>- Each team places 3 objects in their lane end<br>- To reach a goal each team must tip over the opponent team's objects<br>- There is no permanent goalkeeper	**Music:**<br>Music with high BPM to facilitate higher intensity movement.<br><br>**Equipment:**<br>- 6 objects<br>- 1 Soccer ball	**Progression:**<br>- All team members must touch the ball before reaching for a goal<br>- Constant movement, with and without the ball<br><br>**Regression:**<br>- Reduce the number of objects
**Break: 3 minutes rest / active recovery (walking around the gym, moving equipment) / socializing**			
**Circuit training**<br>- 1 x 4 minutes<br>- Intensity (> 85 % HRmax)	- Pick 4-6 exercises (strength-based)<br>- Examples: Jumping Jacks, squats, push-ups, crunches, back extensions etc.<br>- Set a timer 40 sec. of work-out and 20 sec. break + switch	**Music:**<br>Mood music with higher BPM<br><br>**Equipment:**<br>- Stopwatch<br>- Optional: skipping robes, weights, resistance bands etc.	**Progression:**<br>- Longer training sessions before breaktime<br>- Focus on high intensity exercises<br><br>**Regression:**<br>- Shorter training sessions before breaktime
**Cool-down:**<br>- 5-10 minutes<br>- Intensity (40-50 % HRmax)	Stretching<br>Yoga	**Music:**<br>Mood music	

Supplementary table 1:
Comparison between participants with ≥70 % participation in HIIT sessions compared <70 % participation at 3 and 12 months visits. Adjusted for baseline values.

	**Participants (N)**	**Mean difference 3 months (95 % CI)**	**P-value**	**Participants (N)**	**Mean difference 12 months (95 % CI)**	**P-value**
**BMI z-score**	82	0.04 (-0.12; 0.19)	0.66	67	0.002 (-0.17; 0.18)	0.98
**Waist (cm)**	81	0.56 (-4.27; 5.40)	0.82	66	-2.25 (-7.72; 3.21)	0.42
**Systolic blood pressure (mmHg)**	79	-1.93 (-5.73; 1.87)	0.32	66	-1.07 (-5.30; 3.16)	0.62
**Diastolic blood pressure (mmHg)**	79	-0.13 (-2.81; 2.54)	0.92	66	-0.82 (-3.78; 2.13)	0.59
**WHO-5 score**	82	3.73 (-3.38; 10.83)	0.30	63	-0.64 (-8.84; 7:56)	0.88
**PedsQL Child**						
Total score	77	4.29 (0.32; 8.26)	0.03	61	-2.23 (-6.63; 2.18)	0.32
Psychosocial score	77	2.90 (-1.42; 7.22)	0.19	61	-1.80 (-6.54; 2.94)	0.46
Physical score	82	6.62 (1.70; 11.55)	<0.01	64	-2.69 (-8.26; 2.87)	0.34

Abbreviations: SD: standard deviation, BMI: Body Mass Index, WHO-5: WHO-Five Well-Being Index, PedsQL: Pediatric Quality of Life Inventory, version 4.0 Generic Core Scales

Supplementary table 2:
Absolute values for each outcome measure at 3 and 12 months follow-up, divided by randomization group

	3 month follow-up		12 months follow-up	
**Randomization**	**HIIT**	**TCOCT (control)**	**HIIT**	**TCOCT (control)**
**N (%)**	90 (52.0)	83 (48.0)	90 (52.0)	83 (48.0)
**Waist circumference, mean (SD)**	92.4 (13.0)	88.1 (11.8)	92.5 (12.0)	88.5 (12.8)
**BMI z-score, mean (SD)**	2.5 (0.6)	2.2 (0.6)	2.4 (0.7)	2.1 (0.8)
**Systolic blood pressure (mmHg)**	107.9 (11.8)	106.0 (11.0)	108.6 (9.9)	108.3 (10.5)
**Diastolic blood pressure (mmHg)**	66.3 (6.0)	66.4 (6.1)	67.4 (7.2)	67.8 (6.6)
**WHO-5 score**	73.2 (16.2)	70.6 (17.0)	70.7 (18.0)	68.4 (18.7)
**PedsQL Child**				
Total score	85.4 (12.9)	84.3 (11.4)	82.5 (14.6)	89.0 (9.1)
Psychosocial score	76.0 (13.4)	73.6 (11.4)	75.2 (13.2)	74.6 (9.8)
Physical score	79.3 (12.4)	77.4 (10.5)	77.8 (12.7)	79.8 (8.4)
**PedsQL Parents**				
Total score	84.3 (14.3)	80.8 (14.5)	79.9 (15.4)	84.1 (12.4)
Psychosocial score	74.4 (14.8)	73.0 (11.7)	71.3 (14.4)	72.9 (12.4)
Physical score	77.8 (13.6)	75.9 (11.3)	74.3 (13.9)	76.8 (11.6)

Abbreviations: SD: standard deviation, BMI: Body Mass Index, WHO-5: WHO-Five Well-Being Index, PedsQL: Pediatric Quality of Life Inventory, version 4.0 Generic Core Scales
